# Influence of Submicron Fibrillated Cellulose Fibers from Cotton on Hydration and Microstructure of Portland Cement Paste

**DOI:** 10.3390/molecules26195831

**Published:** 2021-09-26

**Authors:** Jing Wu, Qingjun Ding, Wen Yang, Luoxin Wang, Hua Wang

**Affiliations:** 1 State Key Laboratory of New Textile Materials & Advanced Processing Technology, College of Materials Science and Engineering, Wuhan Textile University, Wuhan 430073, China; Wanglx@wtu.edu.cn (L.W.); huawangabc@163.com (H.W.); 2 State Key Laboratory of Silicate Materials for Architecture, College of Materials Science and Engineering, Wuhan University of Technology, Wuhan 430070, China; dingqj@whut.edu.cn; 3 China West Construction Academy of Building Materials, Chengdu 610015, China; youngsun8228@163.com

**Keywords:** cellulose fibers, submicron, cement paste, hydration, microstructure

## Abstract

This paper reports the influence of submicron hydrophilic fibers on the hydration and microstructure of Portland cement paste. Submicron fibrillated cellulose (SMC) fibers was prepared by the acid hydrolysis of cotton fibers in H_2_SO_4_ solution (55% *v*/*v*) for 1.5 h at a temperature of 50 °C. The SMC fibers were added into cement with a dosage of 0.03 wt.%, and the effect of SMC on the hydration and microstructure of cement paste was investigated by calorimeter analysis, XRD, FT-IR, DSC-TG, and SEM. Microcrystalline cellulose (MCC) fibers were used as the contrast admixture with the same dosage in this study. The results show that the addition of SMC fibers can accelerate the cement hydration rate during the first 20 h of the hydration process and improve the hydration process of cement paste in later stages. These results are because the scale of SMC fibers more closely matches the size of the C-S-H gel compared to MCC fibers, given that the primary role of the SMC is to provide potential heterogeneous nucleation sites for the hydration products, which is conducive to an accelerated and continuous hydration reaction. Furthermore, the induction and bridging effects of the SMC fibers make the cement paste microstructure more homogeneous and compact.

## 1. Introduction

Concrete, one of the most widely used man-made materials, has a notoriously low tensile strength and strain capacity, with poor resistance to crack opening and propagation. Cracks can develop when concrete members deform under different degrees of external or internal strain. The driving forces for concrete cracking include the intrinsic contraction associated with cement hydration (such as autogenous shrinkage), plastic shrinkage induced by the exposed environment, and thermal shrinkage as a result of both factors [1,2]. Modern concrete becomes increasingly susceptible to cracking because of its generally higher cement content, reduced water-cementitious (w/cm) ratio, and pozzolanic mineral admixtures (such as slag cement) [3].

The incorporation of different types and sizes of fibers into cementitious composites is a common method to reduce the brittleness of the matrix and increase its durability, which is proportional to the resistance to crack propagation offered by the fibers bridging the matrix that effectively transfer the load [4].

The use of steel fibers makes the concrete members lighter by reducing their sectional dimension and improving their mechanical properties. However, after prolonged use, products made from steel fiber reinforced concrete (FRC) are known to develop cracks and lose their strength, mainly due to the steel’s corrosion [5,6]. Furthermore, because of their large scale, steel fibers have no effect on the original defects that form during the hydration process of cement.

Synthetic fibers have been used with concrete to improve the cracking resistance, especially to reduce plastic shrinkage cracking [7,8]. However, the main issue with synthetic FRC is that the weak interface zone between the fibers and matrix due to the hydrophobicity of synthetic fibers decreases the toughening effect of synthetic fibers on concrete.

Due to their good thermal conductivity, low sensitivity, and high elastic modulus, carbon fibers have also been used in different types of concrete. Carbon fibers improve the flexural strength, toughness, splitting tensile strength, and cracking resistance of concrete [9,10,11]. However, their use frequently involves higher costs and greater energy consumption during the processing of fiber-reinforced cementitious composites.

When compared to the above fibers, cellulose fibers provide many advantages, including their wide availability, renewability, low density, relatively low cost, biodegradability, adequate stiffness and strength, surface roughness, as well as high specific hydrophilicity [12]. The high hydrophilicity and hygroscopicity of cellulose fibers can be attributed to the hydroxyl groups in the cellulose molecular structure, which results in good compatibility between cellulose fibers and cementitious materials [13]. Thus, cellulose fibers have been widely used as alternatives for conventional reinforcement within concrete. It has also been reported that the ductility and strength of cement concrete were markedly improved by using fibers of sisal [14], bagasse [15], jute [16], coconut [17], bamboo [18], fique [19], hemp [20], as well as flax [21] as reinforcing agents [22].

Despite these advancements, there are still several shortcomings that prevent the wider use of natural cellulose chopped fibers in reinforced cementitious materials for engineering: (1) The durability of cellulose fibers in the alkaline cement matrix is a major obstacle that needs to be overcome before the extensive application of cellulose fibers in reinforced cementitious materials can occur, given that the strength of fibers embedded in a cement matrix deteriorates with age [17,23]; (2) Wet swelling and dry shrinkage result in a significant decrease in the fibers’ mechanical properties and an increase in the porosity of the cement matrix [24,25]; (3) It is difficult to achieve homogeneous dispersion of fibers in a concrete matrix [24,25,26,27].

Microcrystalline cellulose (MCC) [28] fibers or particles and nanocrystal cellulose (NCC) [29] are typically prepared from natural cellulose fibers by mechanical or chemical treatments, with the sugar, pectins, and hemicelluloses removed. Therefore, the use of these materials can potentially overcome the previously mentioned shortcomings of natural fiber. They have been used in cementitious composites to enhance the compressive and flexural strength [30,31,32,33], to increase the degree of cement’s hydration [32,34,35,36,37,38] and to mitigate chloride-ion ingress [39]. MCC fibers modified with tetraethyl orthosilicate have been added to cementitious composites to act as a pozzolans, generating additional C-S-H linkages [40]. NCC has been shown to improve the flexural strength of cement composites due to an increase in the degree of hydration, which is possibly aided by a mechanism referred to as short-circuit diffusion [41]. As submicron fibrillated cellulose (SMC) fibers have a size scale just between that of MCC and NCC, their scale matches the size of cement hydration products, which allows them to induce the microstructure formation of cementitious paste, instead of reinforcing and diffusing. Given the primary role of SMC in Portland cement paste systems, we hypothesize that they: (1) provide potential heterogeneous nucleation sites for hydration products; (2) induce cement hydration products to grow on, along and finally embed SMC fibers; and (3) act as a bridge between cement particles.

In this experiment, the SMC fibers were prepared by the sulfuric acid hydrolysis of cotton, and then added into cement paste to induce the growth of calcium silicate hydrates in order to obtain more homogeneous and uniform microstructures. MCC fibers were used as the contrast admixture. The effect of SMC and MCC fibers on the microstructure and hydration of cement paste was investigated using calorimetry, FT-IR spectroscopy, XRD, TG-DSC, 29Si MAS-NMR, and scanning electron microscopy (SEM). From the results of these analyses, the role of SMC fibers on the hydration process and the microstructure evolution of cement paste is discussed.

## 2. Experiment

### 2.1. Materials

**Figure 1:**
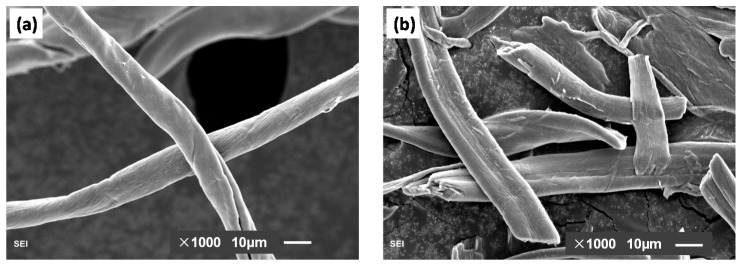
SEM image of (**a**) cotton fibers and (**b**) MCC fibers.

Cotton was used to prepare the SMC fibers. Cellulose microcrystalline (MCC) provided by Shanghai Hushi, Inc. (Shanghai, China) was used as the contrast admixture in this study. Figure 1 shows the SEM image of the cotton fibers and MCC fibers. Concentrated sulfuric acid (98% *v*/*v*) and sodium bicarbonate were purchased from Shanghai Hushi, Inc. Deionized water was used in all experiments. For preparing the cement paste, ordinary Portland cement (OPC) CEM I 42.5, produced by Huaxin cement Co. Ltd. (Wuhan, China), was used with a Blaine fineness of 360 m^2^/kg. Its chemical composition, physical and mechanical properties are shown in Table 1.

### 2.2. Test and Instrumentation

#### 2.2.1. Calorimeter Analysis

A TAM Air isothermal microcalorimeter (NETZSCH, Selb, Germany) was used to measure the heat release rate of the hydrating specimens. The cement, SMC, and MCC were blended with deionized water externally for approximately 1 min, after which the fresh pastes were placed into an airtight glass ampoule and inserted into the calorimeter. The experiment was carried out at a fixed temperature of 20 °C over a period of 72 h.

#### 2.2.2. FT-IR Characterization of Cement Paste

FT-IR spectra were obtained using a JASCO 4100 FTIR spectrometer (JASCO, Tokyo, Japan). The solid pellet samples were prepared by mixing 2–3 mg of sample with 100 mg of KBr.

#### 2.2.3. XRD Analysis

The diffractograms of the samples were determined using a Bruker D8 Advance XRD (Bruker, Berlin, Germany) device with a Cu kα X-ray source at 40 kV and 40 mA (voltage and current, respectively). During data collection, the step-length was 0.02°, the scanning rate was 2°/min and the 2θ range was 5–60°.

#### 2.2.4. DSC-TG Analysis

Thermal analysis (DSC-TG) was conducted using a STA449F3 (NETZSCH, Selb, Germany) apparatus at a heating rate of 15 °C/min from 20 °C to 1000 °C under a nitrogen atmosphere.

#### 2.2.5. Scanning Electron Microscopy (SEM)

SEM (JEOL, Tokyo, Japan) was used to investigate the microstructural properties of the fibers and cement pastes and was performed using an FEI/Quanta450 FEG microscope in secondary electron imaging mode.

### 2.3. Methods

#### 2.3.1. Acid Hydrolysis of Cotton Fibers

**Figure 2:**
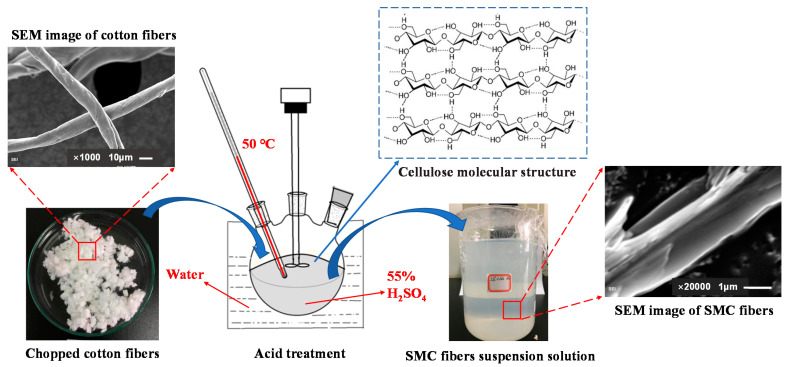
Apparatus for preparation of SMC fibers.

Cellulose fibers typically consist of microfibrils of macromolecules and contain two parts: amorphous regions and crystalline regions. After an acid treatment and high shear mechanical processing of natural cellulose fibers, the amorphous portion of the cotton fibers can be removed or eliminated, allowing submicron-scale SMC fibers that consist of crystalline regions to be prepared. Submicron fibrillated cellulose fibers from cotton can be obtained by optimizing acid hydrolysis process parameters, such as varying acid solution concentration, reaction temperature and time. The apparatus for preparation of SMC fibers is shown in Figure 2.

Cotton was added to 55% (*w*/*w*) sulfuric acid solution and then heated to 50 ℃ with stirring for 1.5 h. After treatment with acid, the entire solution was transferred to a glass beaker containing a twentyfold excess volume of deionized water to terminate the reaction. This solution was kept still until the sediment settled to the bottom of the beaker. The solution supernatant was then removed, and an excess volume of deionized water was added to the beaker. A pH meter was used to measure the pH value of the solution until it was approximately 6.5. This solution was then centrifuged at 4000 rpm to obtain a concentrated aqueous solution of suspended SMC fibers with a solid content of 12–13%, which was used to prepare the samples. An SEM image of SMC is shown in Figure 2, showing that the diameter of the fibers is approximately 1 um. The scale of SMC fibers matches that of cement hydration products.

#### 2.3.2. Preparation of Cement Pastes

Mix proportions are listed in Table 2, and the water/binder ratio of the paste is 0.35. Before the preparation of the mix, MCC and SMC fibers were added into the weighed deionized water and dispersed by ultrasonication, at the power of 325 W and frequency of 20 kHz, for 30 min to obtain uniform suspensions. Cement was added first to the mixing container, and then water containing the dispersed fibers was added into the dry cement and mixed for 3 min with a revolution speed of 60 r/min, then for an additional 2 min with an increased revolution speed of 130 r/min. The cement pastes were cured for 28 days according to GB/T17671-1999 [42], using standard curing conditions at a temperature of 20 °C with a relative humidity of 95–100%. To prepare the samples for SEM, FTIR, XRD, DSC-TG, and NMR, small fragments obtained from the middle portion were placed into an ethyl alcohol solution for 10 days, then dried at 80 °C for 8 h.

## 3. Results and Discussion

### 3.1. Hydration Heat Evolution

**Figure 3:**
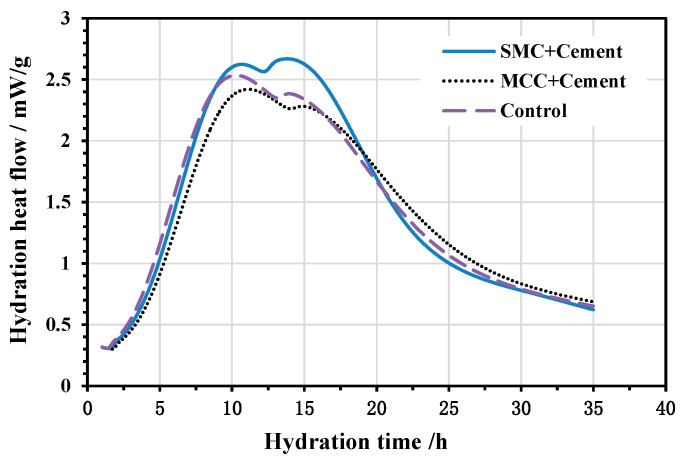
Hydration heat evolution rate curves of cement pastes.

Figure 3 shows the changes in the hydration heat evolution rate of the control paste, the paste with MCC, and the paste with SMC. The acceleration period of each of the three pastes begins approximately 2 h after introduction of the water and ends at approximately 10 h. The MCC and control paste curves are similar in shape, but the SMC curve paste is quite different because its shoulder peak is greater than its main peak. For the MCC paste, there is a tendency for the curve to shift to the right, and the heat evolution rate of the hydration peaks during the acceleration period is lower than the control paste. However, in the SMC paste, the heat evolution rate of the hydration peak is higher than that of the control paste.

Sneillings proposed [43] that the kinetics during the main heat evolution peak are controlled by the growth of C-S-H “needles”, which grow from the surface of Portland cement grains once they are mixed with water. At the heat evolution peak, the surface is covered, but the deceleration is not attributed to diffusion control [44]. The shoulder peak comes from the renewed reaction of C_3_A after depletion of sulfate in solution, but the release of sulfate absorbed onto C-S-H means that ettringite continues to form [45]. Quennoz and Scrivener [46] used in situ XRD to confirm that the formation of ettringite during the shoulder peak occurred around 15 h.

From the results of the hydration heat tests, it can be observed that SMC fibers accelerate the reaction rate during the early hydration period. The reason for this phenomenon is that the primary role of the microscale fibers is to provide potential heterogeneous nucleation sites for the hydration products and to act as growth inducers for the microstructure of cement paste. In contrast, MCC seems to retard the hydration of the cement during the first 20 h; the decrease in the hydration heat flow of sample “MCC+Cement” might be caused by decreasing the water-to-cement ratio because of moisture absorption by the MCC. The hydration heat flow of the MCC sample was higher than that of the SMC and control samples after 20 h, because the water stored in MCC starts to participate in the hydration reaction. Iolanda A. [36] also investigated the effect of microcrystalline and microfibrillated cellulose on the hydration of cement pastes, indicating that the MCC act as water reservoirs. The lack of free water during the hydration process of MCC pastes causes them to release their previously retained water.

**Figure 4:**
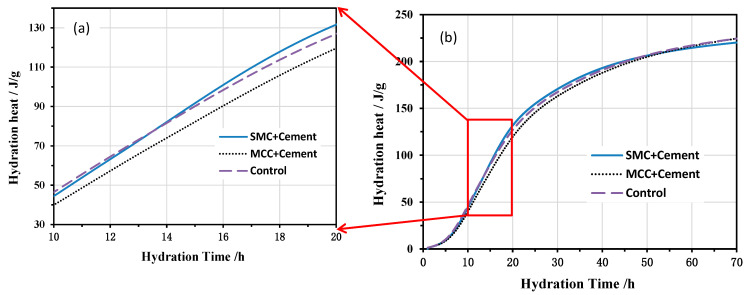
Accumulated heat of hydration for different pastes during (**a**) from 10 to 20 h and (**b**) the first 70 h.

The accumulated heat curves of hydration for different pastes are shown in Figure 4, which shows the relationship between the accumulated heat evolution and the hydration time during 10 to 20 h (Figure 4a) and the first 70 h (Figure 4b). In agreement with the exothermic rate trend, the addition of SMC led to only a slightly higher heat release, whereas that of MCC led to a lower heat release compared to the pure cement paste.

### 3.2. FT-IR Analysis of the Hydrated Cement Pastes

**Figure 5:**
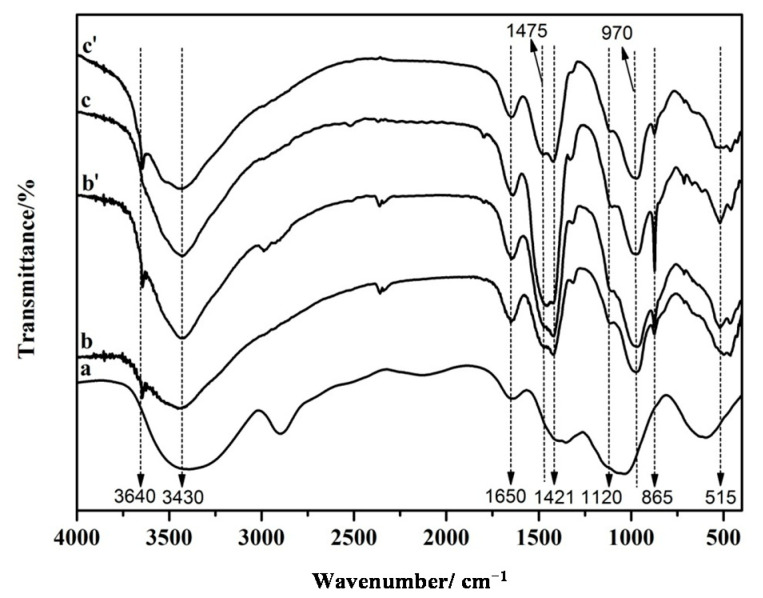
FT-IR spectra of (a) SMC, (b) cement paste at 3 days, (b′) cement paste at 28 days, (c) cement paste with SMC at 3 days, and (c′) cement paste with SMC at 28 days.

The FT-IR spectra of SMC and cement pastes with and without SMC at 3 days and 28 days are shown in Figure 5. The main absorption band and characteristic frequency of cellulose are 610–670, 980–1060, 1421, 1650, 2900, 3200–3450 cm^−1^ [47]. The structure of the SMC is confirmed from the O-H stretching vibration peak at 3350 cm^−1^, the C-H vibration peak at 2900 cm^−1^ and the absorption peak of -CH_2_ on glucose at 1433 cm^−1^. The structure of the C-S-H gel is confirmed from the SiO_4_^2−^ stretching vibration peak at 1175–860 cm^−1^. The Ca(OH)_2_ is confirmed by the O-H stretching vibration peak at 3600–3680 cm^−1^. The AFt and AFm are confirmed by the SiO_4_^2−^ stretching vibration peak at 1120 cm^−1^ and 3640 cm^−1^ respectively. Cement hydration gradually completes as its age increases, with the AFt (3CaO·Al_2_O_3_·3CaSO_4_·32H_2_O) in the cement hydration products transforming into AFm (3CaO·Al_2_O_3_·CaSO_4_·12H_2_O). Therefore, the peak at 3640 cm^−1^ appears in the cement pastes without (b′) and with (c′) SMC fibers at 28 days, but the peak at 1120 cm^−1^ is weakened.

### 3.3. XRD Analysis of the Hydrated Cement Pastes

**Figure 6:**
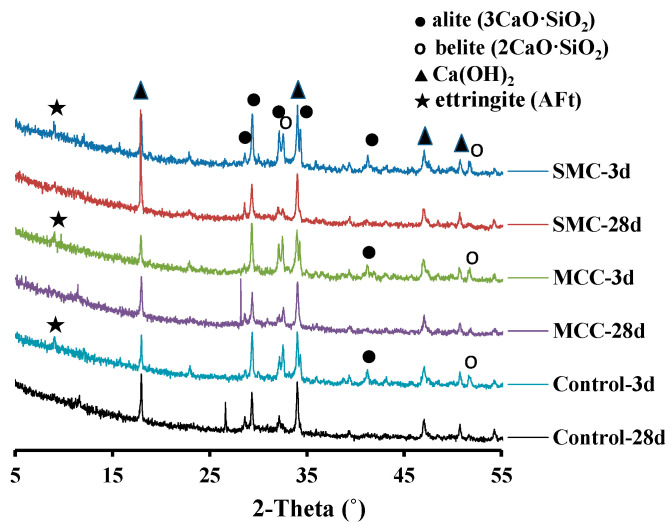
XRD patterns of cement pastes after 3 and 28 days of hydration.

The XRD patterns of different cement pastes at 3 and 28 days are shown in Figure 6. From Figure 6, no obvious new phase is found in the specimens doped with MCC and SMC, and the curves of the control, MCC, and SMC pastes are almost identical at the same age. Results of the XRD analysis indicate the existence of alite (C_3_S), belite (C_2_S), calcium hydroxide (Ca(OH)_2_, CH), and ettringite (AFt). The diffraction peaks of C_3_S and C_2_S in cement at an age of 3 days are still very strong, which indicates that there is some amount of the unhydrated C_3_S and C_2_S present at such an early age. It is worth noting that the diffraction peaks of C_3_S and C_2_S in the SMC sample at 28 days are weaker than that of the MCC sample at the same age. In contrast, it can be clearly seen from the XRD patterns that the diffraction peaks of CH in the SMC sample at 28 days was the strongest. It indicates that SMC contributed to accelerating the hydration of the cement component, which is reflected in the increase in the calcium hydroxide content of hydration products.

The CH peak is the main performance index of cement paste specimens because the CH content in mixtures can be used to track changes in the amount of C-S-H. For the XRD analysis, the main characteristic peak of CH is located at 2θ = 18.05°. Although there is no direct method to determine the concentration of C-S-H, the CH content can indirectly determine the concentration of C-S-H. In order to further explore the reaction mechanism of the three cementitious systems, thermal analysis (TG-DSC) was used to calculate the CH amount in the hydration products.

### 3.4. Thermal Analysis of the Hydrated Cement Pastes

**Figure 7:**
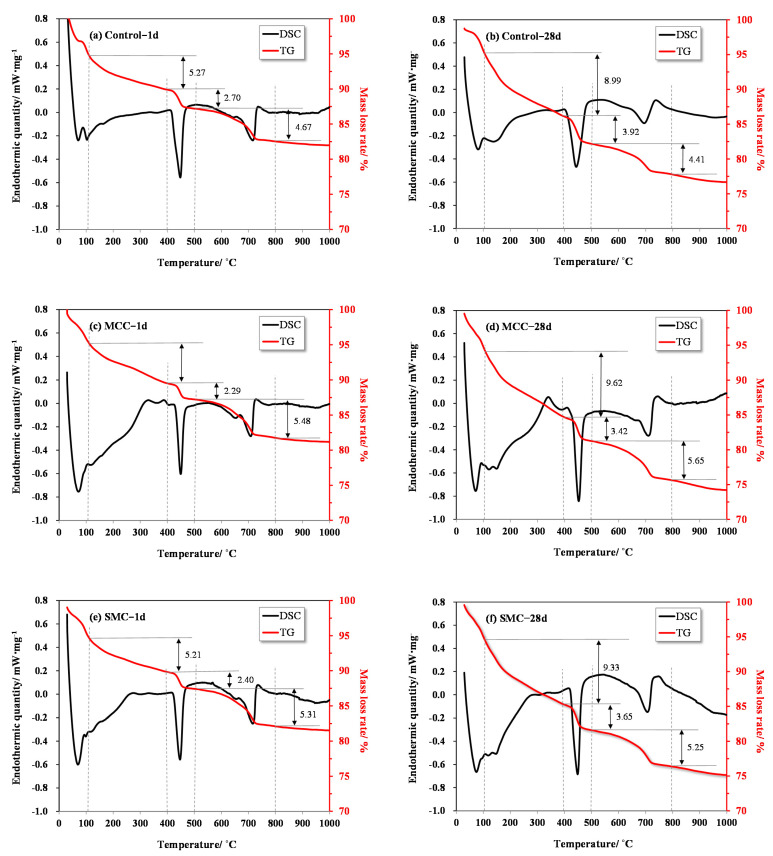
DSC analysis of control pastes and cement pastes with SMC and MCC fibers at the age of 1 day and 28 days.

The thermal analysis (DSC) of samples at 1 and 28 days are shown in Figure 7. The DSC results can be used to determine the crystallinity of CH and other hydrates (water loss), including C-S-H, AFt, gypsum, and others. For those aged 3 and 28 days, the DSC curve of pastes containing SMC were very similar to that of pastes containing MCC, but different from the control sample. After 28 days, the peak of the calcium hydroxide became higher and sharper.

DSC curves exhibit four distinct stages in the decomposition of the control paste, the MCC cement paste and the paste with SMC fibers: (1) at temperatures between 20 and 105 °C, which is related to free water; (2) at temperatures between 105 and 400 °C, which is related to the dehydration of C-S-H gel and decomposition of AFt, AFm and gypsum; (3) temperatures between 400 and 500 °C, which correspond to the decomposition of CH (weight loss *L_CH_*); (4) temperatures between 670 and 780 °C, which correspond to the decomposition of CaCO_3_ (weight loss *L_Calcite_*) [48].

**Figure 8:**
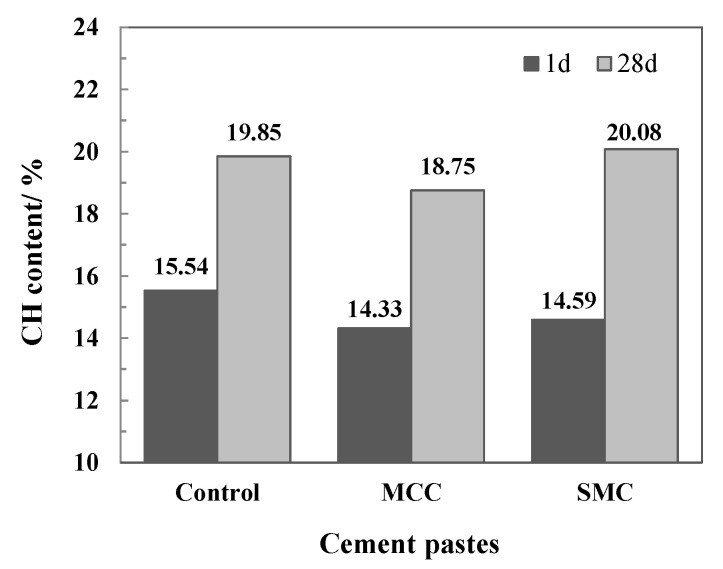
CH content in cement pastes at the age of 1 and 28 days.

For cement paste, the free water content (*W_f_*) and bound water content (*W_b_*, constitutive of C-S-H and AFt) make up the total water content, WT (Equation (1)). Moreover, the total water content (*W_T_*) is calculated as the total weight loss between 20 and 1000 °C (*L_T_*), minus the weight losses due to the dehydroxylation of portlandite (*L_CH_*), the decarbonation of calcite (*L_Calcite_*), and the thermal decomposition of cellulose (*L_Cellulose_*) (Equation (2)). The above data can be obtained from the results of thermal analysis (TG), with the weight loss of cellulose from its content in cement paste (0.03%, Table 2). Furthermore, the total CH content (*P_T_*) of each sample was evaluated using Equation (3). The thermogravimetric analysis results of samples are displayed in Table 3, and the CH content results are shown in Figure 8.

(1) $$W_{T}=W_{b}+W_{f}$$

(2) $$W_{T}=L_{T}-L_{CH}-L_{Calcite}-L_{Cellusose}$$

(3) $$P_{T}=74/18\times L_{CH}+74/44\times L_{Calcite}$$

As shown in Figure 8, the CH contents of the three systems at the age of 1 day are quite close, that of the control, MCC and SMC samples are 15.54, 14.33, and 14.59% respectively. At the age of 28 days, the CH contents of the control, MCC and SMC samples are 19.85, 18.75, and 20.08% respectively. These results indicate that microscale cellulose fibers (MCC and SMC) do not consume CH, because there is no extra pozzolanic reaction caused by MCC and SMC. The SMC cement paste has the highest CH content. These results are in agreement with the analysis of this compound by XRD. It confirms that, compared with MCC, SMC is more conducive to promoting the hydration reaction of cement components, thereby improving the hydration degree of cement. Recent studies indicate that CH formed in pastes containing MCC are more crystalline [36].

### 3.5. SEM

**Figure 9:**
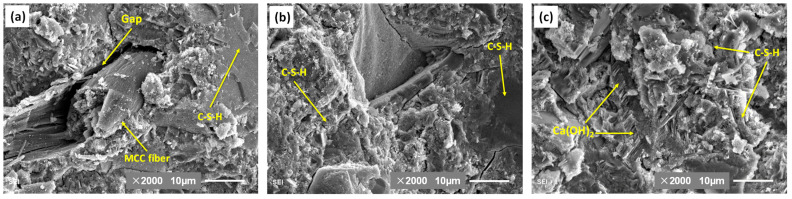
The SEM micrographs of cement paste with (**a**) MCC, (**b**) SMC, and (**c**) control sample at 28 days.

At the early hydration stage, the C-S-H gel gathers around the original cement particles, with the needles on the individual gel clusters interweaving to form a porous structure where they contact one another [49,50]. The properties of high hydrophilicity of cellulose fibers can be attributed to the hydroxyl groups in the cellulose molecular structure, which bring about a good compatibility between cellulose fibers and cementitious materials. The hydration product C-S-H adheres to the surface of the MCC fiber. However, due to the large diameter and the internal empty cavity of MCC fiber, it absorbs water and swells in the early stages. As shown in Figure 9a, with the hydration of cement, the hollow MCC fiber constantly lose water and contract, resulting in gaps appearing around the cotton fiber at the age of 28 days. However, the matrix structure around the MCC is relatively dense.

Figure 9b shows the SEM micrographs of the SMC cement paste at 28 days. It is difficult to find the SMC contour, but the microstructure of cement paste with SMC is more compact and uniform than that of the MCC and control samples. In contrast, the SEM image in Figure 9c shows that the pure cement sample at 28 days has a non-uniform area of its porous matrix, with a large amount of embedded calcium hydroxide. The morphologies of C-S-H gel hydration produced in the three pictures of Figure 9 are obviously different. It is suggesting that inert or active ultrafine particles can promote cement hydration by providing a large surface area for the growth of cement hydration products [51,52,53]. The size effect of SMC fibers induces the microstructure formation of cement paste instead of reinforcing it. It is proposed that the primary role of the submicron-scale fibers is to provide potential heterogeneous nucleation sites for hydration reaction, due to the C-S-H gel growing on and along the surface of the SMC fibers until they are embedded inside of it. The final result is that independent C-S-H gel clusters are connected by SMC fibers, which makes the cement phase microstructure more uniform and compact, contributing to the increased strength and durability of cementitious composites.

## 4. Conclusions

Based on the obtained experimental results, the main results of this study are summarized as follows:

(1) SMC fibers can act as potential heterogeneous nucleation sites for the accumulation of hydration products. Hence, the addition of SMC accelerates the cement hydration rate during the early hydration period.
(2) Compared with SMC, MCC seems to retard the hydration of cement during the first 20 h. The hydration heat flow of samples containing MCC is decreased by decreasing the water-to-cement ratio because of moisture absorption by the MCC. However, the hydration heat flow of the MCC sample increased after 20 h as the water stored in the MCC starts to participate in the hydration reaction. It can be inferred from the CH content obtained from the TG/DTG curves that SMC is more beneficial than MCC to improve the hydration process in later ages.
(3) Since the scale of SMC fibers and C-S-H gels match better than MCC fibers, it is estimated that the nucleation, induction, and bridging effects of SMC fibers make the cement paste microstructure more homogeneous and compact, but further research and advanced material characterization technology is needed to verify this hypothesis.

## Figures and Tables

**Table 1:** Chemical composition, physical and mechanical properties of Portland cement used.

Compound	Mass wt./%	Physical Properties	
SiO_2_	21.70	Starting set time	130 min
CaO	63.01	Ending set time	210 min
Al_2_O_3_	4.76	Surface density	3.01 g/cm^3^
Fe_2_O_3_	3.57	Blaine specific surface	360 m^2^/kg
MgO	2.12	Compressive strength 3 day	35 MPa
SO_3_	1.98	Compressive strength 28 day	62 MPa
Loss on ignition (LOI)	3.5		

**Table 2:** Cement pastes produced.

Mixture Components/wt.%	Control	SMC	MCC
Portland cement	100	100	100
Water	35	35	35
SMC, Submicron fibrillated cellulose fibers	None	0.03	None
MCC, Microcrystalline cellulose fibers	None	None	0.03

**Table 3:** The thermogravimetry results of samples.

Sample	*W_f_*/%	*W_b_*/%	*L_CH_*/%	*L_Calcite_*/%	*L_Cellulose_*/%	*L_T_*/%	*W_T_*/%	Percentage of *W_b_*/*W_T_*/%	Portlandite Content,*P_T_/*%
Control-1d	4.88	5.74	2.70	2.64	0.03	18.02	12.65	61.42	15.54
MCC-1d	4.45	6.56	2.29	2.92	0.03	18.81	13.57	67.21	14.33
SMC-1d	4.00	6.73	2.40	2.81	0.03	18.47	13.23	69.77	14.59
Control-28d	3.63	11.36	3.92	2.22	0.03	23.34	17.17	78.86	19.85
MCC-28d	5.25	11.44	3.42	2.79	0.03	25.79	19.55	73.15	18.75
SMC-28d	4.99	10.94	3.65	3.02	0.03	24.86	18.16	72.47	20.08

## Data Availability

The data presented in this study are available on request from the corresponding author.

## Sample Availability

Samples of the submicron fibrillated cellulose fiber are available from the authors.
